# Transforaminal Lumbar Interbody Fusion (TLIF) versus Oblique Lumbar Interbody Fusion (OLIF) in Interbody Fusion Technique for Degenerative Spondylolisthesis: A Systematic Review and Meta-Analysis

**DOI:** 10.3390/life11070696

**Published:** 2021-07-15

**Authors:** Min Cheol Chang, Gang-Un Kim, Yoo Jin Choo, Gun Woo Lee

**Affiliations:** 1 Department of Physical Medicine and Rehabilitation, Yeungnam University College of Medicine, Yeungnam University Hospital, Daegu 42415, Korea; wheel633@ynu.ac.kr (M.C.C.); chooyj@dgmif.re.kr (Y.J.C.); 2 Department of Orthopaedic Surgery, Hanil General Hospital, Seoul 01450, Korea; 19510035@hanilmed.net; 3 Department of Orthopaedic Surgery, Yeungnam University College of Medicine, Yeungnam University Hospital, Daegu 42415, Korea

**Keywords:** lumbar spine, interbody fusion, oblique lumbar interbody fusion, transforaminal interbody fusion, outcomes, meta-analysis

## Abstract

Preoperative pathology requiring fusion surgery has a great impact on postoperative outcomes. However, the previous clinical and meta-analysis studies did not control for the pathology. In this systematic review, the authors aimed to compare oblique lumbar interbody fusion (OLIF) with transforaminal interbody fusion (TLIF) as an interbody fusion technique in lumbar fusion surgery for patients with degenerative spondylolisthesis (DS). We systematically searched for relevant articles in the available databases. Among the 3022 articles, three studies were identified and met the inclusion criteria. In terms of radiological outcome, the amount of disc height restoration was greater in the OLIF group than in the TLIF group, but there was no significant difference between the two surgical techniques (*p* = 0.18). In the clinical outcomes, the pain improvement was not significantly different between the two surgical techniques. In terms of surgical outcomes, OLIF resulted in a shorter length of hospital stay and less blood loss than TLIF (*p* < 0.0001 and *p* = 0.02, respectively). The present meta-analysis indicated no significant difference in clinical, radiological outcomes, and surgical time between TLIF and OLIF for DS, but the lengths of hospital stay and blood loss were better in OLIF than TLIF. Though encouraging, these findings were based on low-quality evidence from a small number of retrospective studies that are prone to bias.

## 1. Introduction

Achieving solid fusion is paramount for good postoperative outcomes after fusion surgery in the lumbar spine [1]. Interbody procedures with cage insertion are essential for lumbar fusion surgery, and techniques for interbody procedure have advanced over time [2,3,4,5]. Conventionally, cage insertion via the transforaminal route after a posterior approach (transforaminal lumbar interbody fusion, TLIF) has been the gold-standard procedure for interbody fusion; however, the procedure has several drawbacks, including neural injury, endplate violation, cage subsidence, cage migration, and other critical problems [3,6,7,8,9,10,11,12,13,14,15,16,17,18,19,20,21,22,23,24].

Lateral-access cage insertion techniques via the retroperitoneal space have been invented to reduce the problems related to TLIF procedures, including lateral lumbar interbody fusion (LLIF) or oblique lateral interbody fusion (OLIF) [2,4,7,11,12,13,14,15,25,26,27,28,29,30,31,32,33]. LLIF is conducted via the intermuscular approach dissecting the psoas muscle; thus, it can cause psoas muscle-related complications, including anterior thigh pain, leg weakness, nerve root injury, and others [2,4,11,13,14,25,29]. To overcome the problems of LLIF, the OLIF procedure has been recently developed. The OLIF procedure is conducted in the space between the abdominal aorta and psoas muscle, so the risk of psoas muscle injury could be reduced. In addition, several studies have demonstrated that the OLIF procedure provides better outcomes and lower complications than the LLIF procedure [2,4,5,11,14,27,29,30,32,34].

Based on the concepts of the approaches for interbody fusion, several meta-analyses have compared the effectiveness of TLIF and OLIF in interbody fusion [7,8,31,35,36]. However, the previous analyses have had significant limitations: preoperative conditions requiring lumbar fusion surgery varied, such as foraminal stenosis, deformity, instability, and spondylolisthesis. Since preoperative conditions have a great impact on surgical and postoperative outcomes, systematic reviews and meta-analyses should control for these conditions.

In the present study, to accurately compare the outcomes of TLIF and OLIF, we conducted a meta-analysis with suitable previous studies in which only patients with degenerative spondylolisthesis (DS) were studied. Therefore, we aimed to compare surgical outcomes, radiological outcomes, clinical outcomes, and complications of TLIF and OLIF techniques in lumbar fusion surgery for DS.

## 2. Materials and Methods

### 2.1. Search Strategy

This meta-analysis was conducted in accordance with the Preferred Reporting Items for Systematic Reviews and Meta-Analysis guidelines [37]. We systematically searched for relevant articles published up to 8 December 2020 in the PubMed, Embase, the Cochrane Library, and SCOPUS databases. The following keywords were used in the search: “oblique OR transforaminal”, “lumbar spine”, and “fusion”. Filters were used to select studies with human participants.

### 2.2. Study Selection

We applied the following inclusion criteria for the selection of articles: (1) surgical method: posterior lumbar fusion surgery; (2) preoperative condition requiring lumbar fusion surgery: DS; (3) intervention in the experimental group: OLIF technique (OLIF group); (4) intervention in the control group: TLIF technique (TLIF group); (5) study outcomes: radiologic outcome (disc height), clinical outcomes (back pain and radiating pain to lower extremity), surgical outcomes (length of hospital stay, blood loss, and operation time); (6) study design (prospective comparative, retrospective comparative, and randomized controlled studies).

The exclusion criteria were as follows: (1) case reports, reviews, letters, or other undistinctive forms; (2) the same data published repeatedly; (3) study outcomes not reported.

### 2.3. Data Extraction

After discarding duplicate studies, two reviewers independently evaluated the potentially eligible studies. The articles were screened for eligibility based on a review of the title and abstract, and disagreements were resolved through consensus. After screening, the full texts of the eligible articles were read independently by the two reviewers, and the eligibility of each article was re-assessed. Subsequently, the following data were extracted: first author, publication date, study type, number of patients, demographic information (age and sex), type of surgical technique used (OLIF or TLIF), treatment outcome (change in disc height (mm), back pain visual analogue scale (VAS) and leg pain VAS after the operation), and surgical outcomes (hospital stay (days), blood loss (mL), and operation time (min)).

### 2.4. Quality Assessment

The methodological quality of the included studies was evaluated using the Newcastle–Ottawa scale (NOS) [38]. It includes three domains: selection of subjects, comparability of groups, and assessment of outcome. The quality of each study was graded as low (0–3), moderate (4–6), and high (7–9). All divergences were resolved by consensus.

### 2.5. Statistical Analyses

The RevMan 5.3 software program (https://tech.cochrane.org/revman (accessed on 6 July 2021)) was used for statistical analysis of the pooled data. In each analysis, a heterogeneity test was performed using *I*^2^ statistics, which measures the extent of inconsistencies among the results. *I*^2^ = 25% indicated low, 50%, moderate, and 75%, high heterogeneity. *I*^2^ values of ≥50% indicated substantial heterogeneity, and the random-effects model was used for analysis of the data. In contrast, when *I*^2^ was <50%, the pooled data were considered homogenous, and a fixed effect model was applied [39].

To analyze the changes in the disc height, VAS (back), VAS (leg), hospital stay, blood loss, and operation time, we analyzed the standardized mean difference (SMD), which is the difference in the changes in evaluated data after the two surgical operations (OLIF and TLIF). A 95% confidence interval (CI) was used in the analysis. A *p*-value < 0.05 was considered statistically significant.

## 3. Results

### 3.1. Study Selection

**Figure 1:**
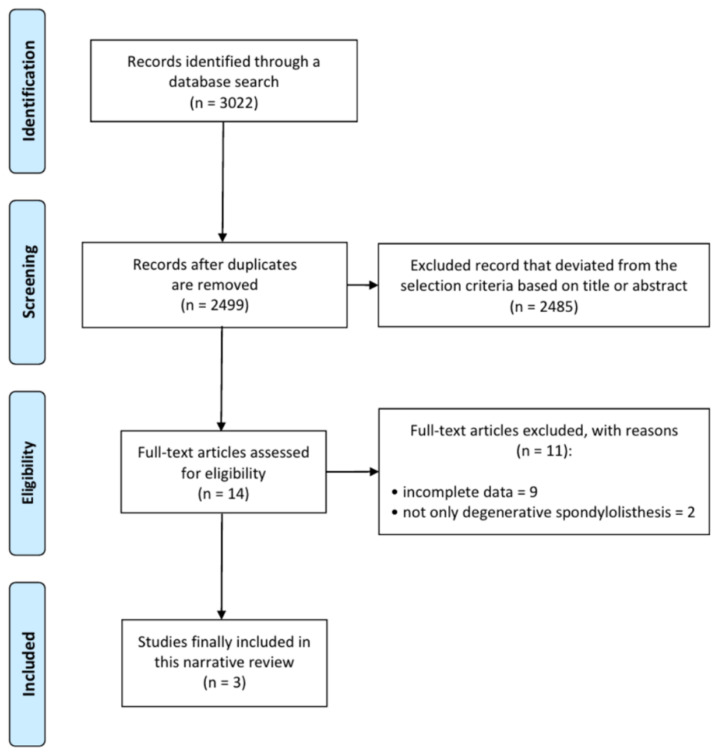
Flow chart showing the search results of the meta-analysis.

A total of 3022 articles were searched, and 523 duplicated articles were removed (Figure 1). After screening for eligibility, based on a review of the title and abstract, 14 articles were selected for full-text reading. After a detailed assessment, 11 articles were excluded due to insufficient results and unsuitable disease type. Accordingly, three studies were finally included in our meta-analysis (Table 1). All included studies were retrospective case–control studies.

### 3.2. Study Characteristics

The three selected studies included 104 cases in the OLIF group and 138 cases in the TLIF group. The detailed characteristics of each study are presented in Table 1.

### 3.3. Risk of Bias

All the included studies were rated out of 9 points (selection of subjects: 4 points; comparability of groups: 2 points; assessment of outcome: 3 points). Therefore, the quality of the four studies assessed using NOS was considered high.

### 3.4. Meta-Analysis Results

**Figure 2:**
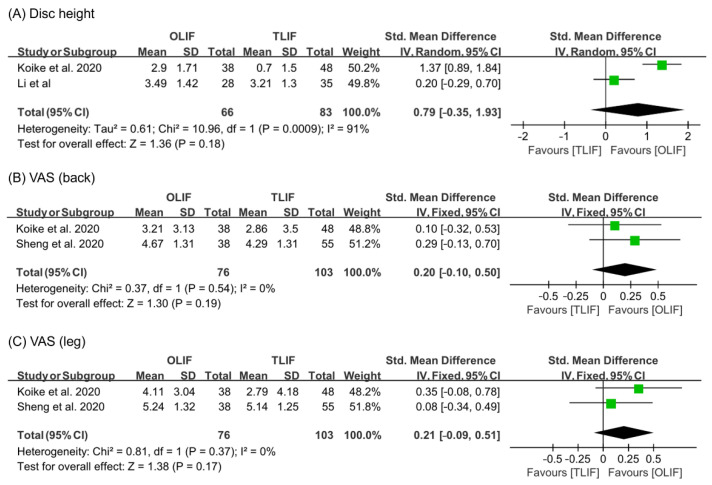
Results of meta-analysis for treatment outcome.

To analyze the changes in disc height, the random effect model was used (I^2^ = 91%). The increase in disc height after OLIF was larger than that after TLIF (SMD = 0.79, 95% CI = −0.35 to 1.93), but there was no significant difference (Figure 2). To determine the change in the degrees of pain in the back and leg, measured using the VAS, the fixed effect model was used (VAS (back): I^2^ = 0%; VAS (leg): I^2^ = 0%). The changes in VAS score for both back and leg were not significantly different between the two surgical operations (VAS (back): SMD = 0.20, 95% CI = −0.10 to 0.50; VAS (leg): SMD = 0.21, 95% CI = −0.09 to 0.51).

**Figure 3:**
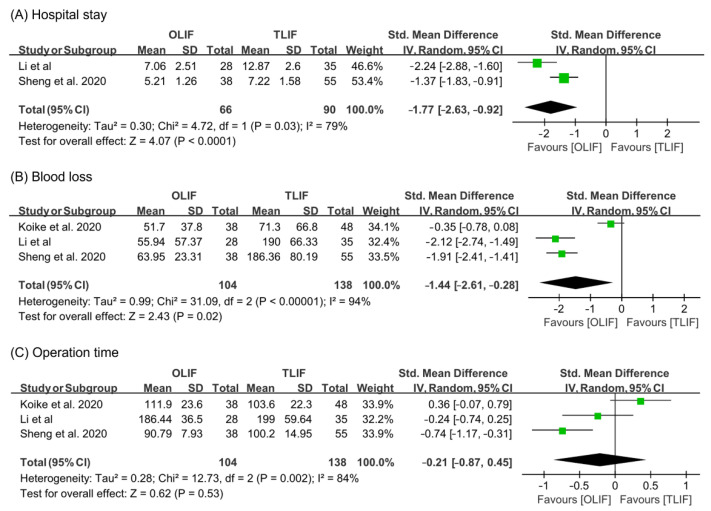
Results of meta-analysis for surgical outcome.

For analyzing hospital stay and blood loss during surgical operation, the random effect model was used (hospital stay: I^2^ = 79%; blood loss: I^2^ = 94%) (Figure 3). OLIF resulted in shorter length of hospital stay and less blood loss than TLIF (hospital stay: SMD = −1.77, 95% CI = −2.63 to −0.92; blood loss: SMD = −1.44, 95% CI = −2.61 to −0.28). However, the results on operation time were not significantly different between OLIF and TLIF (I^2^ = 84%, random effect model, SMD = −0.21, 95% CI = −0.87 to 0.45).

### 3.5. Publication Bias

**Figure 4:**
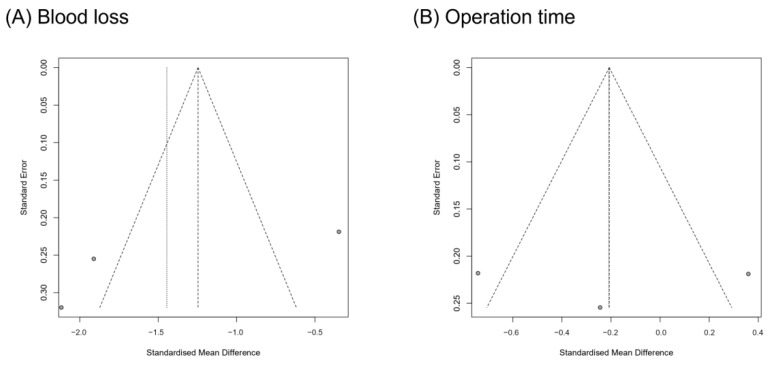
Graphic funnel plots of the included studies.

A funnel plot analysis and Egger’s test were performed for blood loss and operation time (Figure 4). All the *p*-values for the Egger’s test were > 0.05 (blood loss: *p* = 0.358; operation time: *p* = 0.983). Therefore, the publication bias was not significant.

## 4. Discussion

### 4.1. Summary of Lumbar Interbody Fusion Techniques

**Figure 5:**
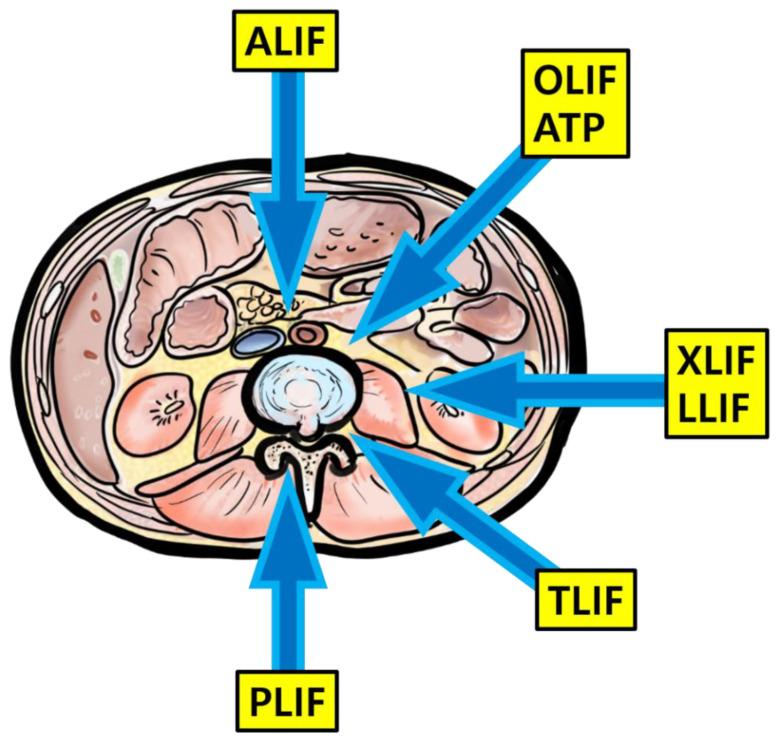
Illustration about lumbar interbody fusion techniques.

Lumbar interbody fusion has been used as a useful surgical treatment option for various lumbar pathological conditions. The main objective of interbody fusion is to restore the intervertebral space and stabilize the segments with proper height and lordosis. Initially, posterior fusion (PLF) was introduced before interbody fusion, but pseudoarthrosis developed after PLF, resulting in an unacceptable incidence. Since the first description of interbody fusion using a posterior approach by Briggs and Milligan in 1944, researchers have developed many other techniques according to their various approaches, such as posterior lumbar interbody fusion (PLIF), TLIF, LLIF, OLIF, and anterior lumbar interbody fusion (ALIF) [2] (Figure 5).

PLIF is a traditional surgical approach for the lumbar spine that allows access to both the anterior and posterior columns with one incision [2,3,4]. Posterior interbody fusion, combined with segmental instrumentation, has become increasingly popular since it was first described by Mercer in 1936 and expanded upon by Cloward [3]. However, PLIF uses a traditional longitudinal midline approach, resulting in inevitably causing paraspinal muscle injury. Additionally, PLIF requires a greater retraction of thecal sac and nerve roots to achieve an adequate surgical exposure of intervertebral space. More recently, the transforaminal approach to the intervertebral disc, known as TLIF, has gained popularity. The basic concept of TLIF is access to the intervertebral disc space from a more lateral trajectory than traditional PLIF; this is generally accomplished through unilateral exposure of the neural foramen and exiting nerve root using a greater degree of facetectomy. Advantages of TLIF over PLIF include better improvement in lumbar lordosis by placement of interbody graft within the anterior column, greater enlargement of the neural foramen, and the option for using an effective unilateral approach; it provides a surgical reservoir for the other aspects of the posterior column integrity, such as the contralateral lamina, facet, and pars, which provide a greater fusion bed for posterior and posterolateral bony arthrodesis. To date, minimally invasive (MIS) lumbar fusion is being increasingly performed, with the clear advantages in the tissue disruption and patient morbidity [4,5,6]. Similarly, MIS-TLIF has been shown to have decreased operative time, intraoperative blood loss, incidence of perioperative infection, and decreased overall hospital stay and earlier return to daily living compared to open TLIF. Patient-reported postoperative outcomes of MIS-TLIF are known as similar or slightly better than open TLIF.

Lateral access to the anterior spinal column has some distinct advantages over traditional posterior approaches, including indirect neurologic decompression with minimized tissue damage, decreased blood loss, and shorter surgical times [6,7,8,9,10]. With LLIF, posterior musculature and ligamentous complexes of lumbar spine are not sacrificed. With larger access space to the intervertebral disc, a more thorough removal of disc material and more efficient end-plate preparation are allowed. Moreover, lateral access enables larger interbody grafts than the posterior approach that can span the apophyseal ring to provide maximum support for fusion. Some disadvantages of LLIF include injury to the psoas muscle, lumbar plexus, or bowel. Approach-sided lower extremity weakness and paresthesia can also occur. Furthermore, researchers have also reported incisional hernia and vascular injury. There are a few things to keep in mind for a successful LLIF procedure. Patient positioning on the surgical table is the cornerstone for an ideal operation. Preoperatively, vascular structure evaluation avoids potential catastrophic complications. On the other hand, OLIF is an alternative to LLIF to overcome its inherent complications. A working corridor for the disc space access of OLIF is placed between the psoas muscle and the aorta to reduce the risk of injury to psoas muscle and lumbosacral nerve plexus. However, the risk of vascular injury can increase because of the proximity of OLIF to the vessels as compared to LLIF. In addition, another complication of OLIF is sympathetic injury because sympathetic chains exit on the working window. OLIF should use a left-side approach because a right-side working passage between inferior vena cava (IVC) and psoas muscle is very narrow, and aorta is more robust to manipulation than IVC. The oblique working corridor is established by the anterior retraction of the peritoneum and slight posterior retraction of the psoas muscle. This surgical oblique view is much more favorable for the surgeons as compared to LLIF. Intraoperative neuromonitoring is not needed because lumbosacral nerve plexus is located within the psoas muscle and OLIF does not split the psoas muscle. Any instruments working in the intervertebral disc space during OLIF should be introduced into the disc with the orthogonal maneuver to place the cage in an ideal position and avoid the injury of contralateral nerve root.

Lastly, ALIF via the ventral approach to interbody fusion has a strong benefit to reconstruct the anterior column of the lumbar spine, with a large fusion bed surface [2]. ALIF has the inherent advantage of restoration of sagittal plane alignment and lumbar lordosis. Additionally, by increasing the neural foraminal height, ALIF may provide indirect decompression of the exiting nerve roots. The well-known complications of ALIF include internal hernia, bowel injury, retrograde ejaculation, venous and arterial thrombosis, and pseudarthrosis. At the stage of planning the ALIF procedure, a history of abdominal surgery or radiation therapy should be investigated. Additionally, major vascular structures around the surgical level should be evaluated with magnetic resonance imaging and/or computed tomography scans preoperatively.

### 4.2. Study Summary and Highlights

Various techniques for interbody fusion, including TLIF and OLIF, have been developed [2,4,12,13,14,29]. Several clinical studies and a few meta-analyses have compared postoperative outcomes and complications between the two techniques, TLIF and OLIF [3,5,6,7,8,14,26,30,31,40,41,42,43,44]. However, previous studies did not control for the preoperative conditions requiring lumbar fusion surgery. To overcome this limitation of previous meta-analyses, the present meta-analysis compared the two interbody techniques, TLIF and OLIF, in lumbar fusion surgery, after adjusting for the preoperative condition of DS. The study outcomes showed that there were no significant differences between the two techniques. In addition, in terms of improvement in back and leg pain, there were no significant differences between the two techniques. Approach-related complication rates were also similar between the two techniques. The outcomes of the current study show that the two options, TLIF and OLIF, could provide similar outcomes after lumbar fusion surgery for DS. However, in terms of surgical outcomes, OLIF resulted in shorter length of hospital stay and less blood loss than TLIF, but they were similar in surgical time. The present meta-analysis indicated no significant difference in clinical, radiological outcomes, and surgical time between TLIF and OLIF for DS, but the length of hospital stay and blood loss were better in OLIF than TLIF.

Radiological outcomes are the primary outcome measures used when deciding the surgical techniques for lumbar fusion surgery, where several factors are considered, including restoration of disc height, achievement of lordosis angle, and fusion rate [1,2,29,45]. In radiologic outcomes, several factors are considered, including a restoration of disc height, an achievement of lordosis angle, fusion rate, and others. Among the factors, one of the most reliable factors on the technique for cage insertion is a disc height restoration [4,5,11,29], since other radiological outcomes may be related to posterior fixation. In our meta-analysis study, the amount of the disc height restoration is not significantly different between the two groups. In OLIF surgery, one of main issues is whether the indirect decompression can be sufficiently achieved with OLIF technique [36]. In the previous studies, the conclusion could not be made because they did not control for the preoperative condition, which could produce significant bias. In contrast, our study adjusted for the preoperative condition of DS. Despite our efforts with confining the preoperative pathology, we could not determine whether one technique is better option for DS in terms of radiologic outcomes. Further studies with extended studies are needed to better confine.

Pain improvement in the back and leg after lumbar spine surgery is also a critical outcome for deciding the surgical technique. Similar to previous studies, the pain outcomes in the present study were not significantly different between the two groups, regardless of the pain location (back or leg). In the included studies, the TLIF group underwent direct decompression of the affected nerve roots, but the OLIF group underwent indirect decompression under restoration of disc height by a cage insertion. Considering these points, the amount of indirect decompression of the affected nerve roots in the OLIF procedure would be comparable to the amount of direct decompression in the TLIF procedure, as the pain generally originates from the compression of the nerve roots.

Another major concern regarding the OLIF procedure is approach-related complications [2,4,6,12,14,29,46]. The OLIF procedure is conducted via the retroperitoneal approach, close to the abdominal vessels, psoas muscle, ureter, and others. Spine surgeons are unfamiliar to these structures, so they are always concerned about the risk of the approach-related complications. In the present study, approach-related complication rates were similar between the two groups. In the previous studies investigating the types of complications generated during the procedure, the TLIF technique had a higher risk of nerve injury, cage migration and subsidence, and hematoma than the OLIF technique. However, the OLIF technique had a higher risk of injury to ureter, major vessels, sympathetic chain, and other retroperitoneal structures than the TLIF technique. Spine surgeons are concerned about OLIF-procedure-related complication, especially injury to major vessels and other critical retroperitoneal structures injury. However, our study showed that the incidence was extremely rare (Table 1), and the rate of major complication was not significantly different between the two groups. Hence, in terms of approach-related complications, the OLIF procedure is comparable to the TLIF procedure, so excessive concern regarding the OLIF technique is unnecessary. Meanwhile, surgical outcomes, such as blood loss, surgical time, and hospital stay, were also analyzed in the meta-analysis. Blood loss and length of hospital stay were significantly less in the OLIF group than in the TLIF group, but the surgical time was not significantly different. Some patients with DS have comorbid illnesses, such as diabetes, cerebrovascular disease, and heart problems, which sometimes lead to poor prognosis and fatal consequences after lumbar fusion surgery because of hemodynamic instability due to blood loss and hypotension. Hence, our study showed that the incidence of major vessel injury was extremely rare, but it is critical for unstable patients if it occurs. Therefore, OLIF may be a good option for patients at high risk of bleeding, but further study is needed.

### 4.3. Study Limitations and Strengths

This study has some limitations. First, the present study did not include all the outcomes after lumbar fusion surgery, due to lack of the information in the literature. Although almost necessary outcomes were addressed and analyzed in the study, some minor factors could not be evaluated in the present study, owing to the insufficient outcomes of the included studies. Second, the number of included studies for our meta-analysis is relatively small. In the future, a meta-analysis compensating these limitations should be conducted. Nevertheless, this study has significant strengths. This was the first meta-analysis, to our knowledge, to compare TLIF and OLIF in lumbar fusion surgery in patients with DS. A few meta-analyses have compared TLIF and OLIF in lumbar fusion surgery but included all kinds of preoperative pathologies, such as foraminal stenosis, deformity, and instability. However, the preoperative pathology can be linked significantly to the postoperative outcomes, which may have caused a significant bias, especially in the previous meta-analyses. Thus, in the present study, we included studies that focused on DS as the preoperative pathology and posterior lumbar fusion surgery for DS. This strategy helped improve internal and external validity.

## 5. Conclusions

Our study compared two interbody fusion techniques, TLIF and OLIF, used in lumbar fusion surgery for DS as a preoperative pathology. The surgical outcome (length of hospital stay and estimated blood loss) was better with the OLIF technique than with the TLIF technique, but pain scores (back and leg), radiological outcomes (disc height), and approach-related complications, and other parameters were not significantly different between the two groups.

## Figures and Tables

**Table 1:** Characteristics of the selected studies.

No.	Study	Design	Subjects (*n*, Age, M:F)	Follow-Up (Months)	Outcome Assessment	Complications
1	Koike et al. (2020) [7]	Retrospective	OLIF: 38, 72.1 ± 11.4 y, 20:18 TLIF: 48, 70.1 ± 11.5 y, 18:30	OLIF: 18.1 ± 8.5 TLIF: 22.5 ± 12.8	Estimated blood loss; operation time; postoperative C-reactive protein level; lower back pain, lumbar function, walking ability, social life, mental health (Japanese Orthopedic Association Back Pain Evaluation Questionnaire); lower back pain, leg pain, leg numbness (VAS); disc height, slipping length (radiographs); degree of stenosis (MRI)	OLIF: 1 superficial injection TLIF: 1 superficial injection
2	Li et al. (2021) [33]	Retrospective	OLIF: 28, 57.5 ± 10.4 y, 7:21 TLIF: 35, 59.3 ± 9.86 y, 8:27	OLIF: >12 TLIF: >12	Length of postoperative hospital stay; estimated blood loss; operation time; length of bed rest (pain (VAS, ODI); disc height, foraminal height, lumbar lordotic angle, pelvic tilt, pelvic incidence, sacral slope (radiographs); complications) *	OLIF: 1 ileus, 1 numbness TLIF: 3 cerebrospinal fluid leakage and root injury
3	Sheng et al. (2020) [31]	Retrospective	OLIF: 38, 65.3 ± 8.9 y, 9:29 TLIF: 55, 60.6 ± 12.4 y, 25:30	OLIF: >6 TLIF: >6	Length of postoperative hospital stay; estimated blood loss; operation time; activity levels (questionnaires); pain (VAS, ODI); satisfaction; motor and sensory function (modified American Spinal Injury Association scale); restoration, alignment and indirect decompression (radiographs and MRI); complications	OLIF: 1 hip flexion weakness, 1 sensory deficit (neural) TLIF: 2 hip flexion weakness, 1 distal motor weakness, 2 sensory deficit (neural)

M, male; F, female; OLIF, oblique lumbar interbody fusion; TLIF, transforaminal lumbar interbody fusion; y, years; VAS, visual analog scale; ODI, Oswestry disability index; MRI, magnetic resonance imaging. * The data were not used because they were measured at <1 year (3 months and 6 months) after the operation.

## Data Availability

Not applicable.
